# Tau Regulates Endoreplication in *Drosophila* Malpighian Tubules

**DOI:** 10.17912/micropub.biology.001956

**Published:** 2026-02-18

**Authors:** Neha Tiwari, Madhu Tapadia

**Affiliations:** 1 Department of Zoology, Banaras Hindu University, Varanasi, Uttar Pradesh, India

## Abstract

Endoreplication drives polyploidy in metabolically active epithelial tissues. Here we show that loss of *Drosophila* Tau increases nuclear size and DAPI fluorescence in principal and stellate cells of the Malpighian tubule, indicative of elevated DNA content. A similar increase is observed in the salivary gland, another polyploid tissue. These findings reveal a previously unrecognized role for Tau in restraining endoreplication in non-neuronal epithelia.

**Figure 1. Loss of Tau affects endoreplication in Malpighian tubules:**
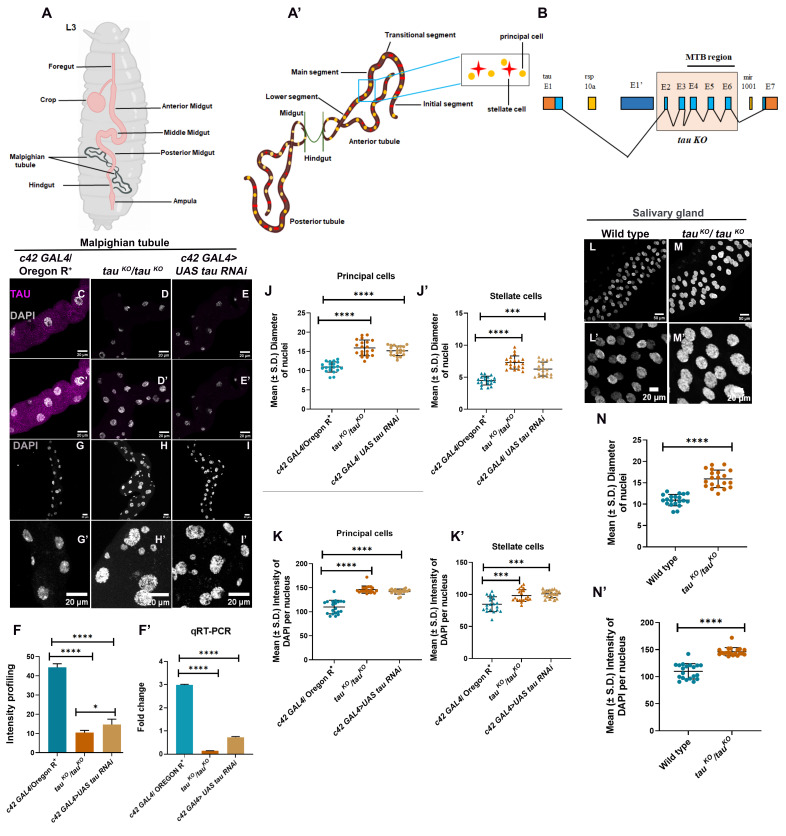
**(A, A’)** Diagrammatic representation of *Drosophila* Malpighian tubules (MTs). **(A)** Larval gut (pink) with MTs shown in green. **(A’)** Classical morphology of larval MTs highlighting principal cells (PCs) and stellate cells (SCs). **(B)** Schematic of the *tau* protein domain organization indicating the region deleted in the *tau ^KO^* allele. **(C–E)** Single-plane confocal images of late third instar MTs stained with anti- *tau* antibody (magenta). **(C’–E’)** Maximum-intensity projection images corresponding to panels (C–E). **(F)** Bar graph showing mean (± SE) fluorescence intensity of *tau* in MTs. **(F’)** Bar graph showing fold change in *tau* transcript levels in third instar MTs. **(G–I)** Confocal images of MTs stained with DAPI (grey). **(G’–I’)** High-magnification views of panels (G–I). **(J, J’)** Quantification (mean, ± SD) of nuclear diameter per nucleus in PCs and SCs (n = 20). **(K, K’)** Quantification (mean ± SE) of mean DAPI pixel intensity per nucleus in PCs and SCs (n = 20). **(L, M)** Confocal images of salivary glands stained with DAPI (grey). **(L’, M’)** High-magnification views of panels (L, M). **(N, N’)** Quantification (mean ± SE) of nuclear diameter and mean DAPI pixel intensity per nucleus in salivary gland cells (n = 20). All images represent late third instar larval MTs and salivary glands. Scale bars: 20 μm (high-magnification MT images), 20 μm (MT overviews), and 50 μm (salivary glands). DAPI stained nuclei (grey). Statistical analyses were performed using one-way ANOVA followed by Tukey’s multiple comparisons test and unpaired t-test. ***p < 0.001; ****p < 0.0001. Error bars represent mean ± SE and ± SD. All images are representative of three or more independent biological replicates.

## Description

Malpighian tubules (MTs) of *Drosophila melanogaster* are specialized epithelial organs responsible for osmoregulation, ion balance, and excretion (Millet-Boureima et al., 2018; Dow and Romero, 2010; Cohen et al. 2020) ( **Figure 1A** ). The MT epithelium contains two major cell types—principal cells (PCs) and stellate cells (SCs)—which differ in both morphology and function ( **Figure 1A ’** ). PCs mediate active cation transport and secretion of metabolites (Terhzaz et al., 2006; Tapadia et al., 2011), while SCs facilitate chloride and water flux (O’Donnell et al., 1983). Both cell types undergo endoreplication, a modified cell cycle involving repeated DNA synthesis without mitosis, generating highly polyploid nuclei essential for the intense transport activity of MT cells (Edgar et al., 2001; Ovrebo and Edgar 2018; Morris et al. 2024). PCs typically reach very high ploidy levels (modal value ~128C), while SCs remain relatively lower (Lamb, 1982).

Tau is a microtubule-associated protein well known for stabilizing microtubules and regulating cytoskeletal dynamics in neurons (Avila et al., 2004; Goedert et al., 2019).

In humans, Tau, encoded by the MAPT gene, is centrally implicated in a group of neurodegenerative disorders collectively known as tauopathies, including Alzheimer’s disease and frontotemporal dementia, where aberrant Tau phosphorylation, mislocalization, and aggregation disrupt cytoskeletal integrity and cellular homeostasis (Avila et al., 2004; Gotz et al., 2019). While Tau pathology has been extensively studied in the nervous system, its functions in non-neuronal tissues, and their potential relevance to disease remain poorly understood.

In *Drosophila* melanogaster, the single Tau homolog—referred to here as *Drosophila tau* represents the ancestral gene from which vertebrate microtubule-associated proteins evolved, with MAPT being the closest functional and evolutionary human ortholog, as supported by FlyBase annotation (Ozturk-Colak et al. 2024) and DIOPT analysis. Similar to its vertebrate counterparts, Tau contains conserved microtubule-binding repeat domains and exhibits microtubule-associated functions (Heidary and Fortini, 2001; Burnouf et al., 2016). Recent studies, including our own work (Valles-Saiz et al., 2022; Tiwari and Tapadia, 2026), show that Tau is also expressed in non-neuronal tissues, yet its physiological roles outside the nervous system remain largely unexplored. Because microtubule stability is directly linked to nuclear architecture, DNA replication, and polyploid cell growth, we hypothesized that Tau may influence endoreplication in epithelial tissues that rely extensively on polyploidization.

To determine whether *tau* is present in the Malpighian tubules, we performed immunostaining with an anti-tau antibody. In control ( *c42 GAL4* / Oregon R ^+^ ) *tau* was detected in the cytoplasm of both principal cells (PCs) and stellate cells (SCs), ( **Figure 1C, C’** ) which can be distinguished by their nuclear size—PCs exhibiting larger nuclei and SCs displaying smaller nuclei. This localization pattern is consistent with the spatial distribution of *tau* across different MT segments that we previously reported (Tiwari and Tapadia, 2026). To investigate the functional requirement of *tau* , we used a *tau* knockout line ( *tau* *^KO^* ), in which exons 2–6 of the *tau* gene is deleted ( **Figure 1B** ) (Burnouf et al., 2016). As expected, immunostaining of MTs from *tau* *^KO^* / *tau* *^KO &nbsp;^* 3rd instar larvae showed a marked reduction in *tau* protein compared with controls ( **Figure 1D, D’** ). This loss was further supported by quantitative fluorescence profiling ( **Figure 1F** ) and a significant decrease in *tau* transcript levels determined by qRT-PCR ( **Figure 1F ’** ).

To complement the genetic knockout, we also performed RNAi-mediated *tau* depletion using the Malpighian tubule–specific driver *c42-GAL4* , which drives expression predominantly in principal cells from early developmental stages (Rosay et al., 1997). Immunostaining of *c42 GAL4> UAS tau RNAi* tubules revealed a reduction in *tau* levels comparable to the *tau ^KO^* condition ( **Figure 1E ’ E’** ), consistent with decreased fluorescence intensity ( **Figure 1F** ) and reduced transcript levels measured by qRT-PCR ( **Figure 1F ’** ). Together, these results confirm that *tau* is expressed in both PCs and SCs and that both the *tau* *^KO^* allele and *c42 GAL4> UAS tau RNAi* efficiently deplete *tau* in the Malpighian tubules.

Having established that Tau is expressed in the Malpighian tubules and is efficiently depleted in both the *tau ^KO^* and *tau RNAi* conditions, we next examined whether loss of *tau* alters nuclear architecture and DNA content in MT cells. Because PCs and SCs are highly polyploid due to endoreplication, changes in nuclear size and DAPI intensity can serve as a direct readout of altered ploidy states. DAPI staining of whole MTs revealed that both the nuclear diameter and DAPI fluorescence intensity were significantly increased in *tau ^KO^* and *c42 GAL4> UAS tau RNAi* tubules compared with controls, indicating elevated DNA content, consistent with increased endoreplication ( **Figure 1G- K’** ). These results suggest that Tau depletion enhances endoreplication or disrupts the regulation of polyploid growth in MT epithelial cells.

To validate that this phenotype is not restricted to Malpighian tubules, we further assessed nuclear morphology in another endoreplicating tissue, the larval third instar salivary gland. Consistent with our MTs observations, *tau ^KO^* salivary glands also displayed enlarged nuclei with increased DAPI intensity relative to controls ( **Figure 1L- N’** ). This cross-tissue concordance strongly supports the idea that Tau plays a broader physiological role in regulating nuclear size and DNA content in polyploid epithelial cells.

*Together, these results identify Tau as a previously unrecognized regulator of nuclear growth and DNA content in polyploid epithelial tissues.*

## Methods

**Fly Stocks**

*Drosophila melanogaster* were maintained on a standard laboratory diet consisting of 10% yeast, 2% agar, 10% sucrose, 10% autolyzed yeast, 3% nipagin, and 0.3% propionic acid. Cultures were kept at 25 ± 1 °C, under 50–70% relative humidity, with a 12:12 h light–dark cycle. The tau knockout (KO) mutant line (Burnouf et al., 2016) was kindly provided by Dr. L. Partridge (Max Planck Institute for Biology of Aging, Cologne, Germany). Dr. J.A.T. Dow (Institute of Biomedical Sciences, University of Glasgow, UK) provided the principal cell-specific Gal4 (c42) driver and *UAS-tau RNAi* (#40875) fly line was obtained from Bloomington&nbsp; *Drosophila* &nbsp;Stock Center (BDSC).

***Drosophila* larval Malpighian tubules, Salivary gland dissection and immunostaining**

Malpighian tubules and salivary glands were dissected from wandering third-instar larvae in chilled 1× PBS. The tissues were fixed in 4% paraformaldehyde for 30 min, followed by three washes in 0.1% PBST (0.1% Triton X-100 in 1× PBS) for 15 min each. After incubation in blocking solution (0.1% Triton X-100, 0.1% BSA, 10% FCS, 0.1% sodium deoxycholate, and 0.02% Thiomersal, in 1XPBS) for 2 h at room temperature, they were incubated overnight in the desired primary antibody at 4°C, following which the tissues were washed three times with 0.1% PBST and incubated in a blocking solution for 2 h. Finally, the tissues were incubated with a secondary antibody at room temperature for 2 h, washed in 0.1% PBST three times, counterstained with DAPI (4',6-Diamidino-2-Phenylindole, Dihydrochloride, Thermo Fisher Scientific, Cat# D1306), and mounted in DABCO (1,4-diazabicyclo [2.2.2] octane, Sigma, Cat# D27802, 2.5% DABCO in 70% glycerol made in 1X PBS) (Tiwari and Tapadia, 2025)

Primary antibodies used for immunohistochemistry was mouse 5A6 Anti-tau (DSHB, 1:20, Cat # 528487 ). The secondary antibodies (1:200 dilutions) was goat anti-mouse Alexa Fluor 647 (Invitrogen). The following stain was used DAPI (1 mg/ml, Thermo Fisher Scientific, Cat# D1306).

**Microscopy and image processing**

Images were acquired on a Zeiss LSM-900 confocal microscope using Zen software (version 3.4) with a 40X objective and identical imaging settings for all tissues. ImageJ software (NIH, USA) was used for image processing. Adobe Photoshop software 2021 (version 22.4.2) was used to assemble the figure panel and schematic diagram were created using Microsoft PowerPoint. **Maximum intensity projections of the confocal z-stacks were used to represent DAPI-stained nuclei.**

**Image quantification and statistical analysis**

Nuclear size and DNA content were quantified from confocal images using **FIJI/ImageJ** . For each condition, images of DAPI-stained Malpighian tubules and salivary glands were acquired with identical acquisition settings to ensure comparability.

Individual nuclei from principal cells (PCs), stellate cells (SCs) and salivary glands were outlined manually using the *Freehand Selection* tool. The *Measure* function was then applied to extract the maximum Feret’s diameter, which served as an estimate of nuclear size. At least 20 nuclei per cell type and genotype were analysed.

Total DAPI fluorescence per nucleus was calculated by multiplying the mean gray value obtained from the Measure function in FIJI by the nuclear area (number of pixels), after background subtraction, and was used as a proxy for nuclear DNA content.

Graphs were plotted using GraphPad Prism 7.0. All statistical analyses were performed using two-tailed unpaired Student’s t-tests for comparisons between two groups, and one-way ANOVA followed by Tukey’s multiple comparisons test for analyses involving more than two groups. Data are represented as mean ± standard error of the mean (SEM) and standard deviation of the mean (SD) ( *n* = 20). *P* values < 0.05 (*) were considered statistically significant.
